# Askin's Tumor: A Dual Case Study

**DOI:** 10.1155/2011/252196

**Published:** 2011-07-18

**Authors:** Bikash Shrestha, Bhupendra Nath Kapur, Kavita Karmacharya, Sunita Kakkar, Ranjit Ghuliani

**Affiliations:** ^1^Department of Pediatrics, Armed Forces Medical College, Pune, India; ^2^Department of Medicine and Oncology, Command Hospital, Southern Command, Pune, India; ^3^Department of Pathology, Armed Forces Medical College, Pune, India; ^4^Department of Pathology, Command Hospital, Southern Command, Pune, India; ^5^Department of Pediatrics, Command Hospital, Southern Command, Pune, India

## Abstract

Askin's tumor is a rare tumor arising from the chest wall. It is a subset of Ewing sarcoma characterized histologically by the presence of small round blue cells. It is a highly malignant tumor with guarded prognosis, which is dependent upon the extension of tumor at the time of diagnosis. A dual paper of Askin's tumors in young boys is being presented here.

## 1. Introduction

Ewing sarcoma comprises of tumors which are characterized by small round blue cells. These include classical Ewing sarcoma of bone, extraskeletal Ewing sarcoma, Askin's tumor of the thoracic wall, and peripheral primitive neuroectodermal tumor [1]. Askin's tumor is a peripheral primitive neuroectodermal tumor of the thoracopulmonary region involving the chest wall [2]. Considering its rarity, we are reporting two cases of young boys who presented with respiratory symptoms and were diagnosed as Askin's tumor subsequently.

**Case 1**

**Figure 1:**
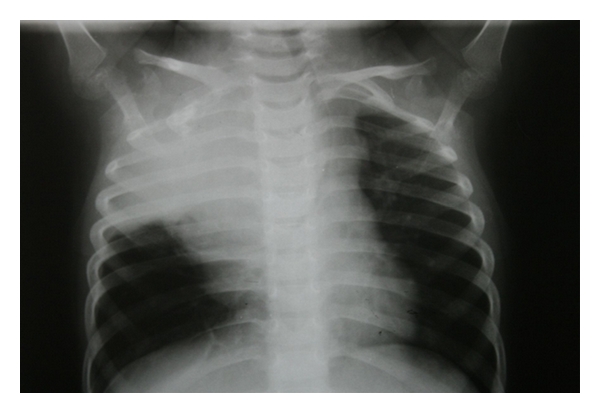
Chest X-ray of Case 1 at the presentation.

**Figure 2:**
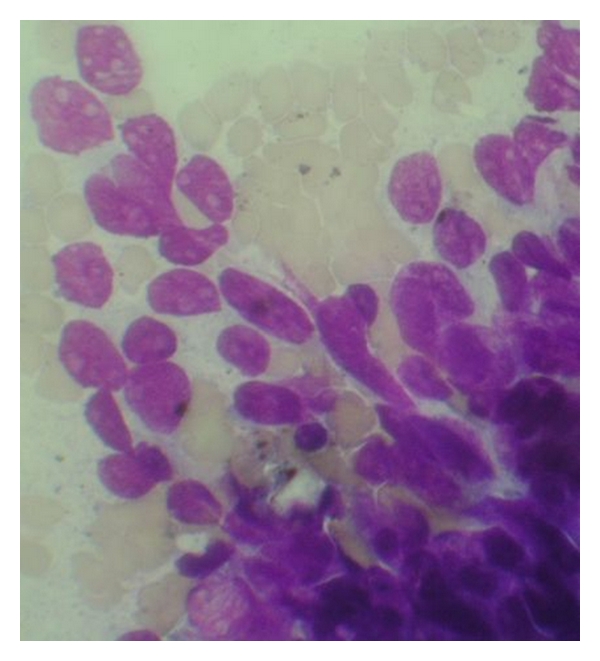
FNAC from the lesion in Case 1 showing the small round blue cells (×400 magnification).

3 years old male toddler, presented with 15 days of cough and 1 day of chest pain, breathlessness, and low-grade fever. He had no other associated complaints, and his past history was unremarkable. Chest radiograph PA view showed a large homogenous opacity on right upper lobe, suggestive of a mass lesion (Figure 1). CT scan revealed a large mass (6.1 cm × 5.6 cm × 5.1 cm) which was merging medially with the mediastinum and laterally, anteriorly, posteriorly and superiorly with the thoracic wall, involving posterior and lateral parts of right 1st and 2nd ribs. Fine needle aspiration cytology (FNAC) from the mass lesion showed small blue round cells with monomorphous salt and pepper appearing chromatin and large and inconspicuous nuclei, suggestive of primitive neuroectodermal tumor—likely Askin's tumor (Figure 2). For diagnostic consideration, biopsy was taken from the mass. It revealed small cells with large nuclei and scanty cytoplasm, with regular chromatin and inconspicuous nucleoli. Considering the site and histology, diagnosis of Askin's tumor was made. He was started chemotherapy with vincristine, adriamycin and cyclophosphamide (VAC), and Ifosfamide and etoposide (IE) alternating 3 weekly cycles as per round cell tumor II (RCT II) protocol [3].

**Figure 3:**
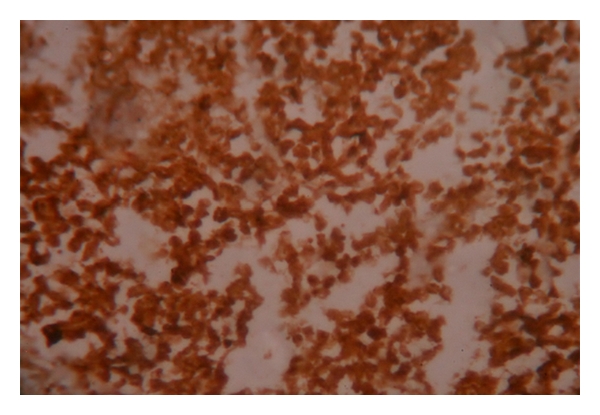
Immunohistochemistry from the lesion showing CK and CD 99 positive in Case 1 (×400 magnification).

The tumor regressed significantly with 8 cycles of chemothearpy. Surgery was undertaken after the course of chemotherapy. Operative findings showed tumor which was arising from the 1st rib, stuck to the apex of the right lung. There was also intense desmoplastic reaction surrounding the tumor, and adhesions were present between the tumor and the right subclavian vessel and superior venacava. Thoracotomy with excision of the tumor and resection of the first rib was done, preserving subclavian vein and brachial plexus. There were no postoperative complications. Postoperative histopathological examination of the mass showed small round blue cell tumors with hyperchromatic nuclei and scanty cytoplasm with tiny foci of calcifications. Immunohistochemistry was strongly positive for CK and CD-99 and negative for LCA and CD-34 (Figure 3).

Postoperatively, he was given ifosfamide, carboplatin, and etoposide (ICE) chemotherapy [4]. Despite adjuvant chemotherapy, he had a relapse after a month of surgery, in the form of right supraclavicular lymphadenopathy. FNAC result from the lymph node was consistent with Askin's tumor. His chemotherapy was then changed to etoposide, vincristine, adriamycin, ifosfamide, actinomycin D (EVAIA) protocol [5]. However, despite 2 cycles of EVAIA, the swelling remained progressive. Considering his poor response, the protocol was changed to cyclophosphamide, vincristine, and dactinomycin (CVD) [6]. He showed response to CVD in the form that his swelling has regressed, but local pain over the operated site has remained persistent. Presently he is undergoing the 6th cycle of CVD and has remained relatively asymptomatic. The plan is to continue CVD cycle for next 12 cycles and reassess further.

**Case 2**

**Figure 4:**
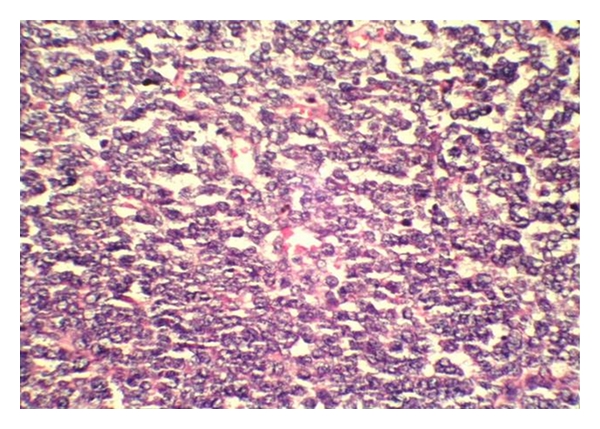
Histopathlogical examination of the biopsy in Case 2 showing sheets of small round blue cells (×400 magnification).

9-year-old boy had presented with chest pain and fever of 7 days duration. There was no history of breathlessness, cough, hemoptysis, or weight loss. His past history and family history were unremarkable. Radiograph of the chest PA view showed large homogenous opacity covering almost the entire right hemithorax. CT scan chest delineated a large mass (9.5 cm × 4.5 cm × 7.3 cm) along the right posterolateral chest wall which was causing scalloping of the inner surface of the 8th rib with obliteration of the extrapleural fat in places with free well defined and irregular border medially. FNAC from the lesion showed small round blue cells, with scanty cytoplasm and relatively large nuclei. Biopsy of the lesion showed small round blue cells in sheets with ill-defined rosette-like structures, histomorphologically peripheral neuroectodermal tumor-possibly Askin's tumor (Figure 4). Immunohistochemistry of the tumor cells was CD-99, synaptophysin, chromoganin focal positive, and desmin, SMA, CK, and LCA negative.

**Figure 5:**
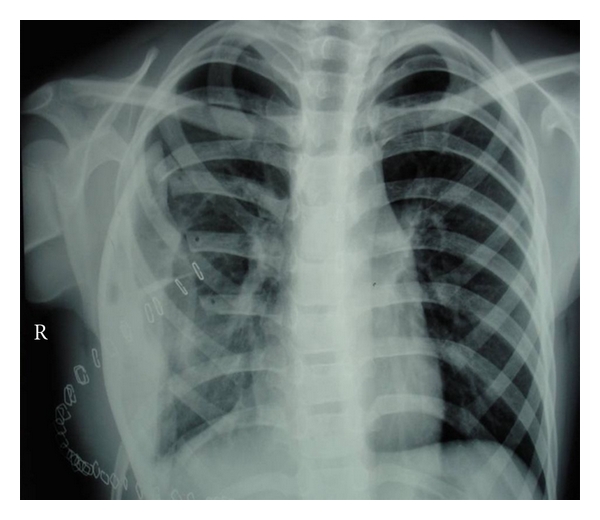
Chest X ray postsurgery in Case 2.

He was given chemotherapy with vincristine, adriamycin and cyclophosphamide (VAC), alternating with ifosfamide and etoposide (IE) as per RCT II protocol [3]. After completion of 4 cycles, the mass showed significant regression. Postchemotherapy, he was taken up for surgery. Per operatively the tumor mass was seen on parietal pleura, extending from the 5th to the 8th ribs and adherent to the chest wall. Wide local excision with en bloc excision of the 5th, 6th, 7th, and 8th ribs was done, leaving behind a clear 2 cm healthy margin. The resected region was reconstructed with bone cement and prolene mesh. The chest radiograph PA view postsurgery is shown (Figure 5). After successful surgery, he was put on alternating cycles of vincristine, adriamycin and cyclophosphamide (VAC) alternating with cyclophosphamide, vincristine, and dactinomycin (CVD). Presently, he has completed 6 cycles of chemotherapy and has remained relapse-free and asymptomatic. We plan to continue chemotherapy to complete 12 cycles and reevaluate further then.

## 2. Discussion

Askin's tumor is a subset of Ewing's sarcoma which arises from the chest wall. It is characterized histologically by features of small round blue cell tumors. It was described by Askin et al. in 1979 [7]. It is more frequent in males than in females (1.5 : 1). Ewing sarcoma is the second most common malignant bone tumor in childhood and sixth most frequent malignant bone tumor. Ewing sarcoma is a rare tumor with incidence of 2.9 per million in populations younger than 20 years and it rarely presents in adults more than 30 years [8–10].

Askin's tumor is genetically defined by reciprocal translocation t(11 : 22) (q24 : q12) with EWS-FLI-1 fusion gene [1, 11, 12]. It is characterized as grey white tumor with various necrotic, hemorrhagic or cystic parts in gross section. Histologically, it is distinguished by typical small round blue cells of monomorphous appearance. It is believed to be of neural crest origin [4, 7–9, 13]. 

Askin's tumor usually presents with common respiratory symptoms. It may include cough, chest pain, fever, breathlessness, and so forth. Local pain may be present which might reduce in intensity at night but not disappear completely. It may present with local paraesthesia and rarely as pathological fracture or metastasis related symptoms [1, 9]. In the cases presented, the manifestation was purely like any lower respiratory tract infections. High index of suspicion is required in such scenario. However, poor response to antibiotics, any associated alarming features and relatively poor general condition of the patient should hint towards more sinister underlying pathology. In doubtful cases, it would be more prudent to investigate aggressively and rule out any serious condition.

Radiography of the chest in Askin's tumor may show classical onion-peel appearance of the tumor or just homogenous opacity [1, 9]. In both of our cases, it was just homogenous opacity. Magnetic resonance imaging is superior to computed tomography scan in delineating the mass accurately because of its intrinsic high-contrast resolution, multiplanar capability, and because of its allowance of a clear assessment of chest wall muscle involvement [14]. Non-Hodgkin's lymphoma, small cell osteosarcoma, metastatic neuroblastoma and so forth, should be considered as differentials during histological study [1, 9, 15]. Immunohistochemistry is used to differentiate and confirm the diagnosis of peripheral neuroectodermal tumors from other round cell tumors [8–11]. 

The treatment includes chemotherapy, radiotherapy and surgery. Surgical therapy has the most important implication; however, because of its position, surgery has to be individualized. Complete surgical resection is associated with a survival advantage [1, 9]. Overall, 5-year survival rate is 70%, prognosis being dependent upon the tumor extension at diagnosis. In our cases here, the clear surgical resection was not possible in the first case due to the anatomical complexities of the involved structures. The relapses which the first case had could be contributed perhaps to the residual tumor which may have been left during the surgery. However, the second case, which had wide local excision of the tumor, showed better response in the form of no relapses so far. However, the cases need to be followed up. Ewing sarcoma is known to be radiosensitive, but raodiotherapy has to be individualized, especially in younger age group, considering the chest wall deformity of the growing bones as well as neurolodevelopmental complications which may be associated with radiotherapy. Radiotherapy was avoided in our cases in view of the younger age group. Postoperative adjuvant chemotherapy and radiotherapy are employed to achieve local control, depending upon the surgical as well as metastatic status. Important complications during treatment include relapses and musculoskeletal abnormalities postsurgery [9]. 

## 3. Conclusion

Askin's tumor is a rare tumor of childhood which usually presents with common respiratory symptoms. Pediatrician must be far sighted to suspect this rare entity with such common presentations. It would require a high index of suspicion on behalf of the general pediatrician for the early recognition of this tumor so that chances of better outcome and prognosis shall be there. However, considering its aggressive nature, complicated course and recurrence tendency, long term followup is warranted.
